# The Need for a Mental Health Technology Revolution in the COVID-19 Pandemic

**DOI:** 10.3389/fpsyt.2020.00523

**Published:** 2020-06-03

**Authors:** Caroline A. Figueroa, Adrian Aguilera

**Affiliations:** ^1^School of Social Welfare, University of California, Berkeley, Berkeley, CA, United States; ^2^Department of Psychiatry, Zuckerberg San Francisco General Hospital, University of California, San Francisco, San Francisco, CA, United States

**Keywords:** mobile health (mHealth), COVID-19, apps and smartphones, telemental health, public mental health, psychological therapies, depression, anxiety

## Introduction

The current coronavirus 2019 (COVID-19) pandemic not only poses a large threat to the physical health of our population, if we fail to act now, it will also have detrimental long-term consequences for mental health.

Though social distancing is a crucial intervention to slow down the destructive effects of the pandemic, it can lead to isolation, decreased physical activity, and increased rumination, which might particularly hurt those with pre-existing mental illness. Further, the stream of disheartening COVID-19 news provides fodder for increased worry and distress, which can be detrimental for people with anxiety disorders. Early cross-sectional surveys in the United States, Canada, and Europe show an increase in symptoms of depression and anxiety for the general population, associated with COVID-19 concerns (1). Thus, this crisis is exacerbating existing mental health conditions and creating conditions for the development of new ones. Further, if lessons from other outbreaks such as Ebola (2) and SARS (Severe Acute Respiratory Syndrome) (3) are any indication, even after an outbreak is controlled, there will likely be a substantial increase in need for psychological support.

Crucially, this public health crisis will magnify and deepen existing shortcomings of mental health care systems. The US was already facing a mental health crisis before the pandemic: less than half of those with mental illness receive the care that they need (4). Underserved populations, such as low-income or ethnic minority populations, are disproportionately affected; they show the lowest utilization of mental health services (5). Early data suggest that underserved populations pay a larger health toll from COVID-19: they show higher mortality rates (6, 7). They are more likely to work in essential jobs putting them at greater risk of contracting COVID-19, and suffer greater economic consequences. All these factors lead to increased stress and anxiety. We will therefore be faced with an even greater relative shortage of trained professionals and means to mental health care during and after this pandemic.

We argue that what we need during a public health crisis like this is a digital mental health revolution: scaling up the delivery of confidential mental health services to patients across a wide range of platforms, from telemental health to mobile interventions such as apps and text messaging. Here, we provide an overview of technological tools which could help to decrease the mental health burden of COVID-19, provide recommendations on how they could be used and scaled-up, and discuss considerations and limitations of mental health technology applications.

### Telehealth

There is a crucial role for the use of teleconferencing software for therapy sessions during the COVID-19 pandemic. Most studies of teleconferencing services showed that effectiveness is comparable to in-person services across disorders including depression, posttraumatic stress disorder, and anxiety disorders (8). China has had some success with this approach. Researchers recently wrote in a Lancet Commentary that during the worst of the outbreak in January, China successfully provided online psychological counseling and self-help was widely rolled out by mental health professionals in medical institutions, universities, and academic societies (9).

In the US, the pandemic has also catalyzed a rapid adoption of telehealth (10). Medicare now allows for billing for telehealth. Further, the Health Insurance Portability and Accountability Act (HIPAA) has been revisited to permit more medical providers to use HIPAA compliant platforms to communicate with patients. This removes a major barrier to wider adoption of telemedicine and could also provide an outstanding opportunity for patients who previously did not feel comfortable seeking mental health care to now approach these services.

However, it is important to attend to disparities in technology access and digital literacy. Before the pandemic, only one in ten patients in the US used telehealth, and 75% said that they were unaware of telehealth options or how to access it (11). Recent data from primary care clinics showed that, though video care consults went up by 80% in late March and early April, minority groups represented a smaller portions of these visits (12). This is partly explained because of a lack of Internet availability, which varies due to limited data plans and lack of Wi-Fi, and inability to use smartphone features such as downloading apps (13). At the moment, some US telecom providers are offering free Internet services (14). However, longer-term strategies need to be developed to prevent further widening of the digital divide (15), including providing affordable, high speed Internet access, improving usability of telehealth programs, and providing appropriate guidance/training for patients using these services.

### Mental Health Smartphone Applications

Importantly, the use of personal mobile phones presents an opportunity for broad scaling of interventions. Over 90% of Americans have some type of mobile phone and over 80% have smartphones (16). Even among low-income Americans (71%) and older adults (53%) smartphone ownership is high. Mental health apps have shown effectiveness in decreasing symptoms of depression (17) and anxiety (18). Because of COVID-19, multiple meditation and wellness apps designed by the private sector have now temporarily opened up free memberships to aid in easing anxiety, the majority of these being mindfulness apps (19).

However, there are over 10,000 consumer-available mental health apps in app stores and many of these are not evidence-based (20). Further, though many people download mental health apps, research shows low rates of continued use over longer periods of time (21). It is crucial that mental health providers recommend apps that are backed up by evidence. One helpful resource is Psyberguide (www.psyberguide.org), a non-profit that rates apps based on the strength of the scientific research that supports it, ease of use, and its privacy policies (22). Lastly, in order to improve engagement, providers should follow up with patients on their usage of these apps and integrate the app content into their treatment.

### Texting Applications

In addition to apps, text-messaging platforms could be leveraged to help people cope with mental health challenges evoked by COVID-19. Because texts are also delivered *via* individuals' devices, they are easy to provide to many at once using automated text-messaging platforms. Text-messaging interventions have demonstrated effectiveness in behavioral health promotion and disease management (23). Importantly, text-messaging is an appropriate tool for low digital literacy populations and underserved groups (24). For instance, our own HIPAA approved texting platform, HealthySMS, was developed with and for low-income populations (mostly Spanish speakers) and shows high acceptability in underserved populations (25). We recently rolled-out a text-messaging study to provide wide-scale support to interested individuals in the US *via* daily automated text-messages, containing tips on coping with social distancing and COVID-19 anxiety.

For crisis situations, Crisis Text Line provides free confidential help *via* text-message. This platform has seen the mention of “coronavirus” in 24% of conversations from March 30th to April 6th (26). Furthermore, Caremessage, a non-profit organization, has temporarily provided free access to their messaging platform and COVID-19 template text-messaging library with health information (27). In addition, reliable information can also be delivered by health and government organizations automated *via* text messages. Scaling of information delivery to patients and the public could also relieve health professionals and public health departments, who are already understaffed, underfunded, and overburdened (14).

### Social Media

Social media plays a complicated role in the management of mental health. On the one hand, it can provide positive and supportive connections during a time of physical isolation. Earlier work shows that many people with mental illness are increasingly turning to social media to share their experiences and seek mental health information and advice (28). On the other hand, it can also serve to increase depression and anxiety symptoms based on negative social comparisons and the spread of distressing information (29). For instance, in a recent cross-sectional survey of almost 5,000 participants in China, increased social media exposure on COVID-19 was associated with increases in anxiety and depression symptoms (30).

Social media has played a large role in the spread of information since the start of the COVID-19 outbreaks, including misinformation and “fake news”. Large social media platforms are now reportedly taking steps to remove false content or conspiracy theories about the pandemic, using artificial intelligence (AI); and distribute reliable information, such as developed by the World Health Organization (31).

In China, the government provided online mental health education through popular social media platforms, such as WeChat, Weibo, and TikTok during the height of the outbreak in January (9). In the UK, the National Health Service (NHS) is working with Google, Twitter, Instagram, and Facebook to provide the public with accurate information about COVID-19 (32).

Social media also provides a unique opportunity for health professionals to distribute accurate information to their patients and the public, or to highlight available mental health resources. In Wuhan China, mental health professionals uploaded videos of mental health education for the general public through WeChat and other Internet platforms at the early stage of the outbreak (9). In the US and Europe, many physicians have turned to Twitter to share medical information. The social media site has now implemented a mechanism to verify physicians and other scientific experts in an effort to counteract coronavirus misinformation (33).

However, because of the overload of information on social media, misinformation might still spread too fast to be intercepted by AI algorithms (34, 35). A recent report of responses from more than 8,000 people from six countries showed that one third reported seeing a significant amount of false or misleading COVID-19 information on social media or messaging platforms (36).

Further, posting information on social media raises the question of how health professionals should respond to the information posted by patients, and how that can impact the therapeutic relationship. Currently, there are no clear guidelines for health professionals, to determine how to act on social media. This calls for a push in quickly establishing such a consensus (37).

## Discussion

The COVID-19 crisis has fast-forwarded the use of technology in mental health care. Technology is crucial in scaling up access to mental health services during and after COVID-19. Given that people interact differently with technology, people of various ages, technical abilities, languages, and levels of literacy will need distinct types of interventions (38).

Older people are particularly vulnerable during this pandemic and already suffer from high rates of loneliness (39). This is strongly associated with greater symptoms of depression and anxiety (40), and physical morbidities and mortality (41). Previous work shows that older adults are interested in using technology to support their mental health, and that mobile health technology is feasible and reliable for assessing cognitive and mental illness (42).

However, older adults and those with low digital literacy might lack prior knowledge of digital technology to fully benefit from these tools (43). Digital health tools suffer from usability issues: they do not always consider digital literacy, health literacy, age, or English proficiency in their design (44). For instance, previous work showed that even the most basic functions of apps are difficult to use for diverse populations (45). Top-funded digital health companies test only 30% of their apps in people with clinical conditions (46). These factors are important because individuals with lower health literacy have worse health outcomes over time due to difficulty making informed health choices (47).

Therefore, interventions should be specifically targeted toward vulnerable groups, and adapted to their specific needs. This includes design choices such as easy to navigate user interfaces and tailoring vocabulary to older adults or those with low English proficiency (48). Training for individuals with low-tech skills, through outreach programs by healthcare staff may help patients to understand and use digital tools (49). Health systems should prioritize implementation of this crucial service (12).

Further, the right infrastructure needs to be set up to provide digital interventions securely, without personal privacy violations and minimizing the risk of data breaches. Apps and text-messaging must not only be effective, but also safe, secure, and responsible, similar to how therapists are held to standards of responsible practice and confidentiality (42). Therefore, it is imperative that cybersecurity specialists also become involved in ensuring safe technological services (50). Finally, just as they have now shown flexibility with telehealth, insurance companies and health systems should begin covering digital and mobile health interventions.

## Conclusion

We are now in the midst of an acute health crisis which calls for a grand upscaling of mental health resources. Technology provides a medium for delivering mental health services remotely and on a wide scale, which is particularly important during social distancing measures. Even when the worst of the COVID-19 pandemic has subsided, it is likely that a large need for mental health support and services delivered through technology will remain. Digital mental health tools should be affordable, accessible, and appropriate for a wide group of individuals with varying ages, languages, and digital literacy. The time to massively invest in high quality and accessible online and mobile mental health in the face of the COVID-19 pandemic, and possible future pandemics, is now.

## Author Contributions

CF wrote the first draft of the article. AA contributed to the writing and editing of the manuscript. Both authors contributed to the editing of the final manuscript.

## Conflict of Interest

The authors declare that the research was conducted in the absence of any commercial or financial relationships that could be construed as a potential conflict of interest.
